# Valproate inhibits mitochondrial bioenergetics and increases glycolysis in *Saccharomyces cerevisiae*

**DOI:** 10.1038/s41598-020-68725-5

**Published:** 2020-07-16

**Authors:** Michael Salsaa, Bianca Pereira, Jenney Liu, Wenxi Yu, Shyamalagauri Jadhav, Maik Hüttemann, Miriam L. Greenberg

**Affiliations:** 1 Department of Biological Sciences, Wayne State University, Detroit, MI 48202 USA; 2 Center for Molecular Medicine and Genetics, School of Medicine, Wayne State University, Detroit, MI USA; 3 Present Address: Department of Human Genetics, University of Michigan, Ann Arbor, MI USA; 4 Present Address: Genetics and Metabolism Section, Liver Diseases Branch, National Institute of Diabetes and Digestive and Kidney Diseases, National Institutes of Health, Bethesda, MD USA

**Keywords:** Biochemistry, Medical research

## Abstract

The widely used mood stabilizer valproate (VPA) causes perturbation of energy metabolism, which is implicated in both the therapeutic mechanism of action of the drug as well as drug toxicity. To gain insight into these mechanisms, we determined the effects of VPA on energy metabolism in yeast. VPA treatment increased levels of glycolytic intermediates, increased expression of glycolysis genes, and increased ethanol production. Increased glycolysis was likely a response to perturbation of mitochondrial function, as reflected in decreased membrane potential and oxygen consumption. Interestingly, yeast, mouse liver, and isolated bovine cytochrome *c* oxidase were directly inhibited by the drug, while activities of other oxidative phosphorylation complexes (III and V) were not affected. These findings have implications for mechanisms of therapeutic action and toxicity.

## Introduction

Bipolar disorder (BD) is a severe psychiatric illness characterized by shifts in mood, ranging from mania to depression. It affects at least 1% of the population and leads to suicide in 15% of cases^1^. BD patients exhibit a higher prevalence of obesity, cardiovascular disease, and diabetes than the general population^2,3^. Many studies have shown that the pathophysiology of BD involves altered energy metabolism^4–8^. While most of the metabolic markers measured indicate mitochondrial dysfunction in BD^9–12^, some studies have suggested the presence of increased mitochondrial activity in the manic phase of BD^13^, including increased energy generation^14^, basal metabolic rate^15^, uric acid^16^, and calcium ions^17^.

Valproate (VPA) is one of several mood stabilizers approved by the FDA for the treatment of BD^18,19^, epilepsy^20,21^, and migraine^22^. The mechanism of action of VPA is not understood^23^. VPA is effective in only 40–60% of cases and can cause serious side effects, including hepatotoxicity and teratogenicity^24^. Hepatotoxicity can be life-threatening^25,26^ and may occur even at therapeutic doses^27^. Although rare, lethal hepatotoxicity associated with VPA has been described in both children^28^ and adults^29^. The prominent feature of this type of hepatotoxicity is microvesicular steatosis^30^, consistent with mitochondrial dysfunction^31^. In agreement with this, patients with congenital defects in mitochondrial metabolism are at a higher risk for susceptibility to VPA toxicity^32–34^.

VPA exerts numerous documented effects on mitochondrial metabolism. It is metabolized by and inhibits ß-oxidation through several mechanisms^35^. VPA and its metabolites sequester coenzyme A (CoA), depleting mitochondrial CoA^36^. Furthermore, studies suggest that both unesterified VPA as well as VPA acyl-CoA esters inhibit fatty acid oxidation enzymes^37,38^. In addition to affecting ß-oxidation, VPA inhibits α-ketoglutarate dehydrogenase, a key enzyme of the tricarboxylic acid (TCA) cycle^39,40^. Inhibition of this enzyme is a proposed mechanism underlying decreased TCA cycle flux in the presence of VPA^41^. VPA also decreases levels of carnitine^42,43^, which transports fatty acids into the mitochondria. Other effects of VPA on mitochondrial energy metabolism include a decrease in pyruvate uptake^44,45^ and inhibition of mitochondrial oxidative phosphorylation^44,46,47^. Komulainen et al*.* reported that VPA decreases the oxygen consumption rate (OCR), mitochondrial membrane potential (ΔΨ_m_), and ATP levels in hepatocytes (HepG2) after 48 h of treatment^48^. While these studies indicate that VPA inhibits mitochondrial metabolism, the resulting consequences for other metabolic pathways have yet to be established.

The aim of the current study was to investigate the ramifications of VPA-induced inhibition of mitochondrial bioenergetics using the yeast model, in which genetic and biochemical analyses of the metabolic effects of VPA have been uniquely informative^49,50^. In a previous screen of the yeast deletion mutant collection to identify mutants exhibiting sensitivity to VPA, several mutants with defects in mitochondrial functions were identified as hypersensitive to the drug^49^. In addition, a microarray analysis of yeast gene expression revealed that VPA altered the expression of many metabolic genes and increased the expression of several glycolytic genes^50^. In the current study, we report that VPA decreases mitochondrial OCR and ΔΨ_m_ and increases levels of glycolytic metabolites. Interestingly, VPA inhibited cytochrome *c* oxidase (COX) activity in yeast and mammalian cells, while activities of complex III or V were not affected. These findings suggest that the metabolic effect of VPA is an increase in glycolysis most likely to compensate for the inhibition of mitochondrial bioenergetics.

## Results

### VPA increases glycolytic activity

**Figure 1:**
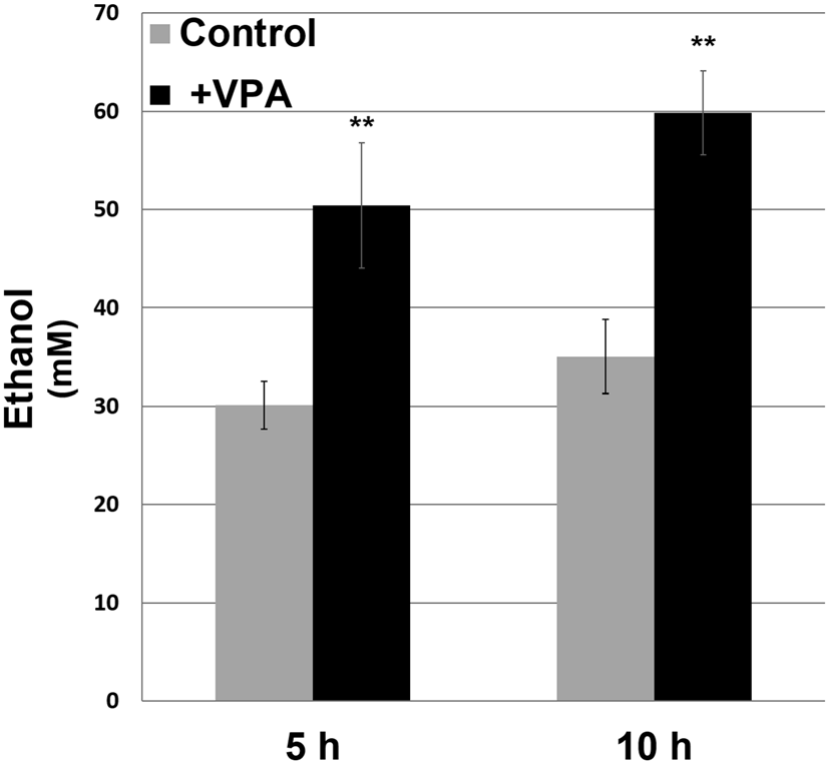
VPA increases ethanol production. Cells were cultured in the absence or presence of VPA (0.6 mM) for 5 or 10 h, harvested, and ethanol was assayed in supernatants by enzyme-coupled fluorescence assay (BioVision). Ethanol concentration is in mM ethanol normalized to cell density measured at A_550_. Data shown are mean ± SD (n = 3) (**p < 0.01).

We previously conducted a genome-wide microarray analysis to identify pathways affected by VPA^50^. Cells grown in the presence of VPA (0.6 mM) for 5 h exhibit increased expression of glycolytic genes (Table 1). Consistent with an increase in glycolysis, we observed a significant increase in ethanol production after 5 h and to a greater extent after 10 h (Fig. 1).

**Table 1:** VPA up-regulates expression of glycolysis genes.

Enzyme name	Gene name	Fold change	Standard error
Hexokinase isoenzyme 2	*HXK2*	4.2	0.47
Alpha subunit of phosphofructokinase	*PFK*	2.3	0.3
Beta subunit of phosphofructokinase	*PFK2*	2.1	0.24
Triose phosphate isomerase	*TPI1*	2.4	0.31
Phosphoglycerate mutase	*GPM1*	2.3	0.29
Enolase 1	*ENO1*	2.7	0.39
Enolase 2	*ENO2*	3.2	0.38
Enolase related repeat/functional repeat	*ERR2*	3	0.43
Enolase related repeat/functional repeat	*ERR3*	3	0.36

Mid-log phase (A_550_ = 0.5) cells were cultured in the presence or absence of VPA (0.6 mM) for 5 h, total RNA was extracted, and mRNA levels were determined by microarray analysis and analyzed using Gene Spring software. Statistical significance was calculated using student’s *t*-test with a p-value less than 0.05 for all genes. Fold change reflects expression in cells grown in the presence of VPA relative to vehicle-treated control cells^50^.

**Figure 2:**
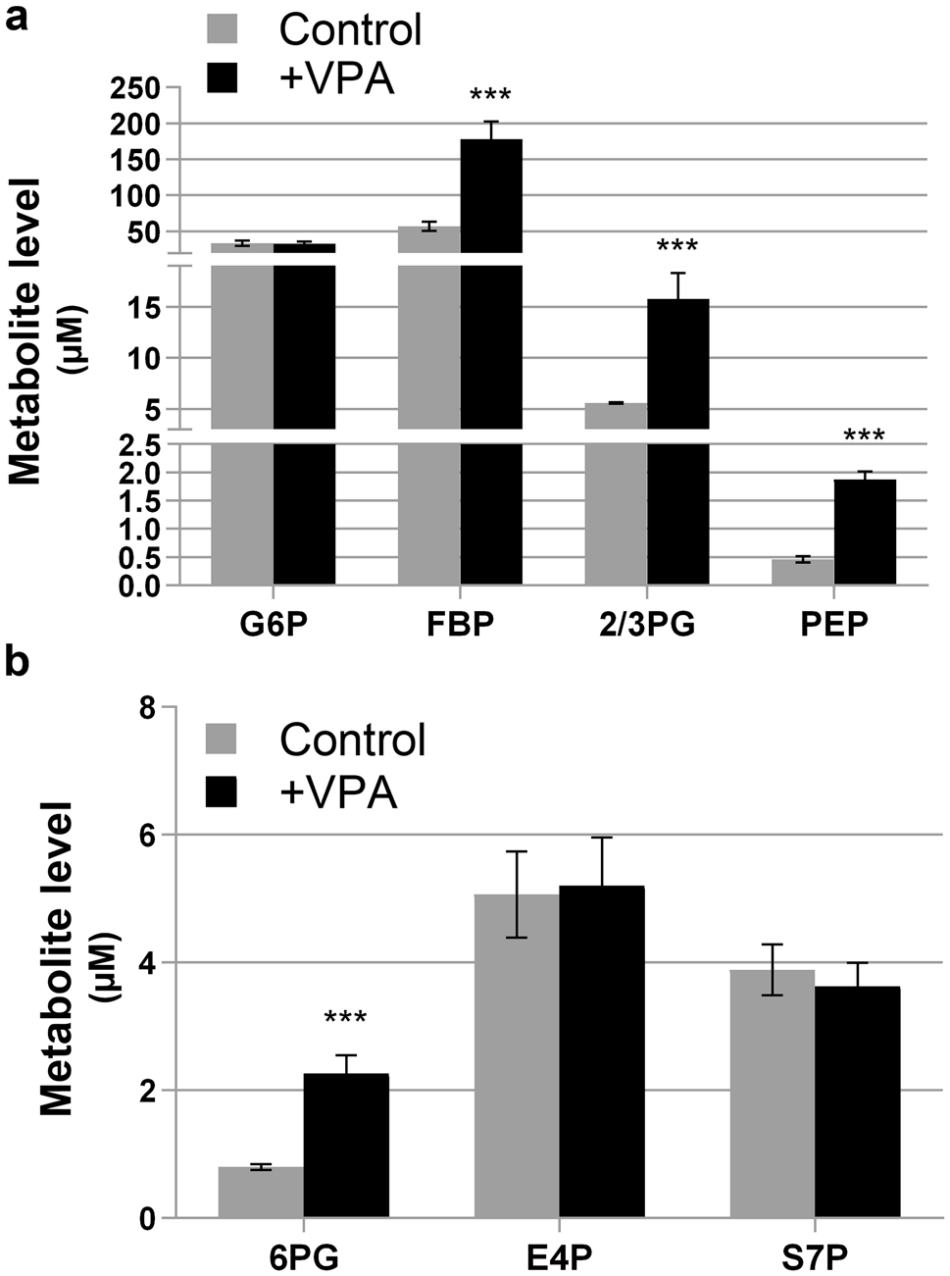
Effect of VPA on steady state levels of glycolysis and pentose phosphate pathway intermediates. Cells were cultured until the mid-log phase and treated with VPA (1 mM) for 5 h, lysed, and metabolites were determined by mass spectrometry. The values represent the concentration of each metabolite (µM) normalized to cell density measured at A_550_. Data shown are mean ± SD (n = 4) (***p < 0.001). (**a**) Glycolysis. (**b**) Pentose phosphate pathway. *G6P* glucose-6-phosphate; *FBP* fructose-1,6-bisphosphate; *PG* phosphoglycerate; *PEP* phosphoenolpyruvate; *6PG* 6-phosphogluconic acid; *E4P* erythrose-4-phosphate; *S7P* sedoheptulose-7-phosphate.

An increase in carbon flux through glycolysis and/or a decrease in carbon flux to acetyl-CoA due to inhibition of mitochondrial activity can lead to an increase in ethanol production. To test the possibility that VPA increases glycolysis, cells were treated with VPA and levels of glycolytic intermediates were determined by mass spectrometry. VPA-treated cells exhibited increased levels of glycolytic intermediates fructose-1,6-bisphosphate, 2-phosphoglycerate/3-phosphoglycerate, and phosphoenolpyruvate compared to untreated cells (Fig. 2a). These findings indicate that VPA increases carbon accumulation through the glycolytic pathway, which explains the observed increase in ethanol production.

An increase in 6-phosphogluconic acid, which is part of the oxidative phase of the pentose phosphate pathway, was also observed. However, metabolite levels in the non-oxidative phase of the pathway (erythrose-4-phosphate and sedoheptulose-7-phosphate) were not affected (Fig. 2b). This may demonstrate an increased need for NADPH, which is generated only during the oxidative phase. VPA increases reactive oxygen species levels and induces the oxidative stress response in yeast^51,52^. Therefore, cells may require increased NADPH, which is utilized to reduce glutathione that is essential to neutralize reactive oxygen species.

**Figure 3:**
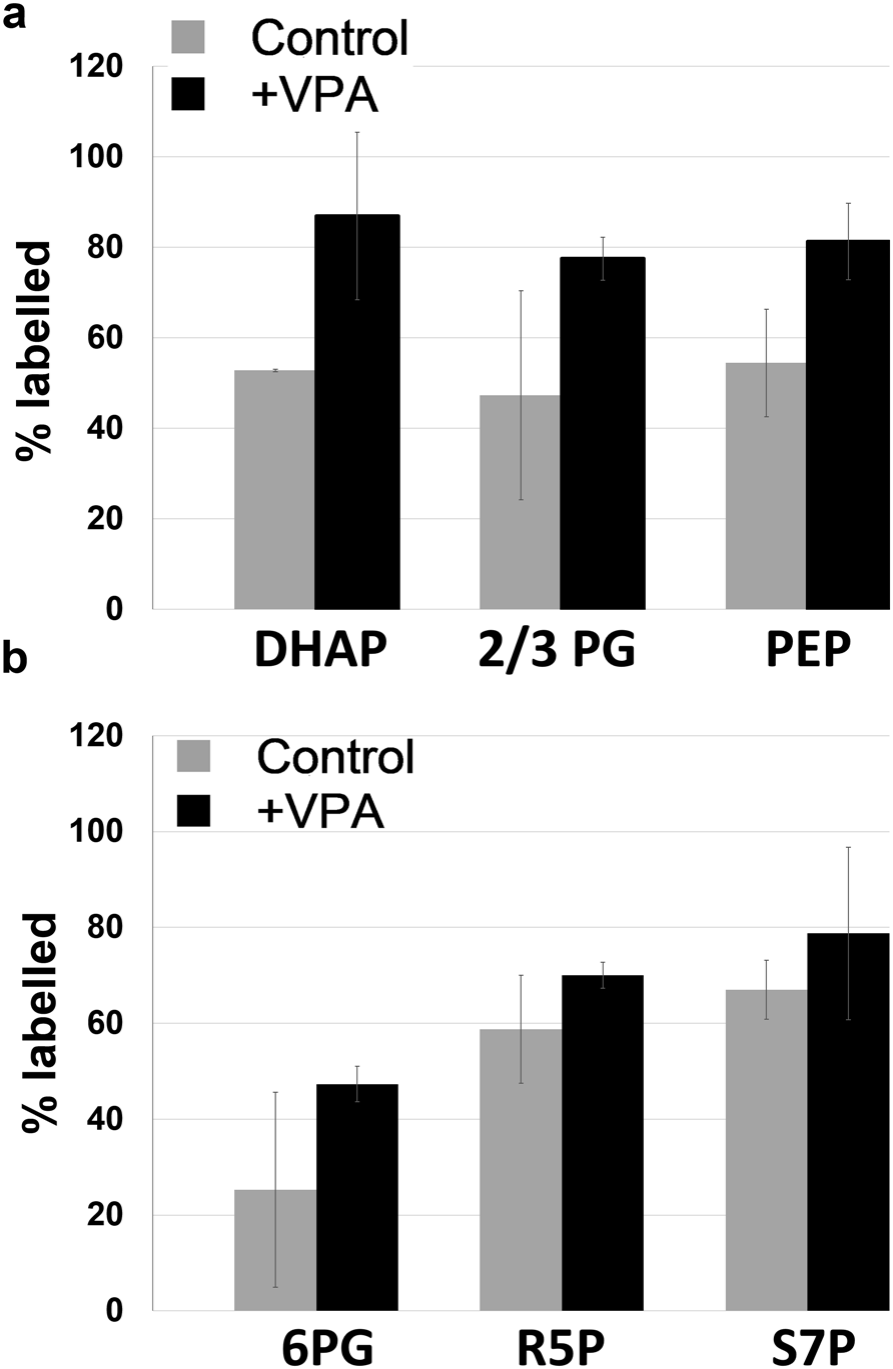
Effect of VPA on [U-^13^C]-glucose metabolic labelling through glycolysis and the pentose phosphate pathway. Cells were grown to the mid-log phase, treated with VPA (0.6 mM) for 4.5 h, harvested, then incubated in fresh medium containing [U-^13^C]-glucose for 20 min. Cells were then washed with ammonium acetate and snap frozen. To assay carbon flux, cells were lysed, protein concentration was determined, and quantification of [U-^13^C]-labelled glycolytic and pentose phosphate pathway metabolites was determined by mass spectrometry. The values shown (% labelled) represent the percentage of each metabolite uniformly labelled with ^13^C. Data shown are mean ± SD (n = 2). (**a**) Glycolysis. (**b**) Pentose phosphate pathway. *DHAP* dihydroxyacetone phosphate; *PG* phosphoglycerate; *PEP* phosphoenolpyruvate; *6PG* 6-phosphogluconic acid; *R5P* ribulose-5-phosphate; *S7P* sedoheptulose-7-phosphate.

In further support, a metabolic labelling experiment utilizing [U-^13^C]-glucose suggests that VPA increased carbon flux to several glycolytic intermediates (Fig. 3a) and to 6-phosphogluconic acid (Fig. 3b), which can explain the accumulation of these metabolites described above.

### VPA decreases mitochondrial bioenergetics

**Figure 4:**
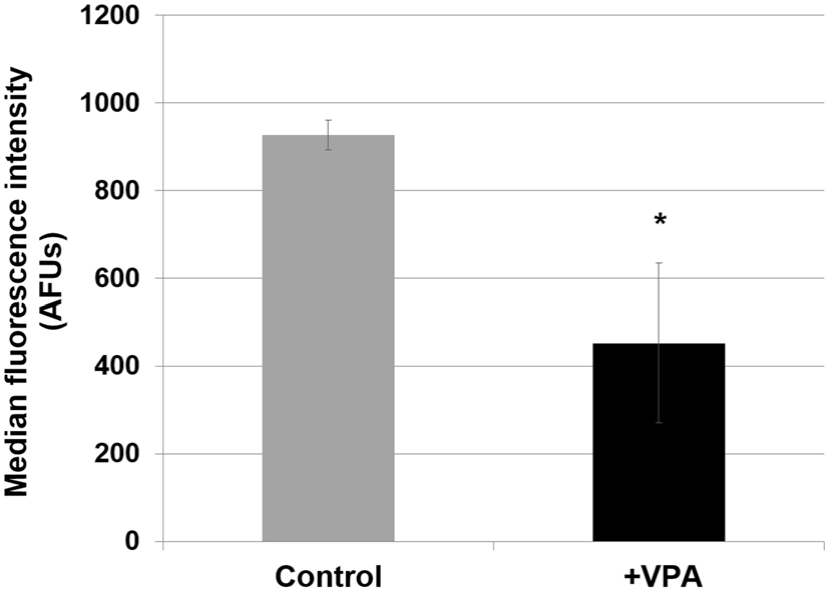
VPA decreases mitochondrial membrane potential. Cells were grown to the mid-log phase, incubated with VPA (0.6 mM) and TMRM for 1 h, washed once, and fluorescence was measured using flow cytometry. Median fluorescence intensity of single cells was analyzed with FlowJo. Data presented are in arbitrary fluorescence units (AFUs) and represent mean ± SD (n = 3) (*p < 0.05).

**Figure 5:**
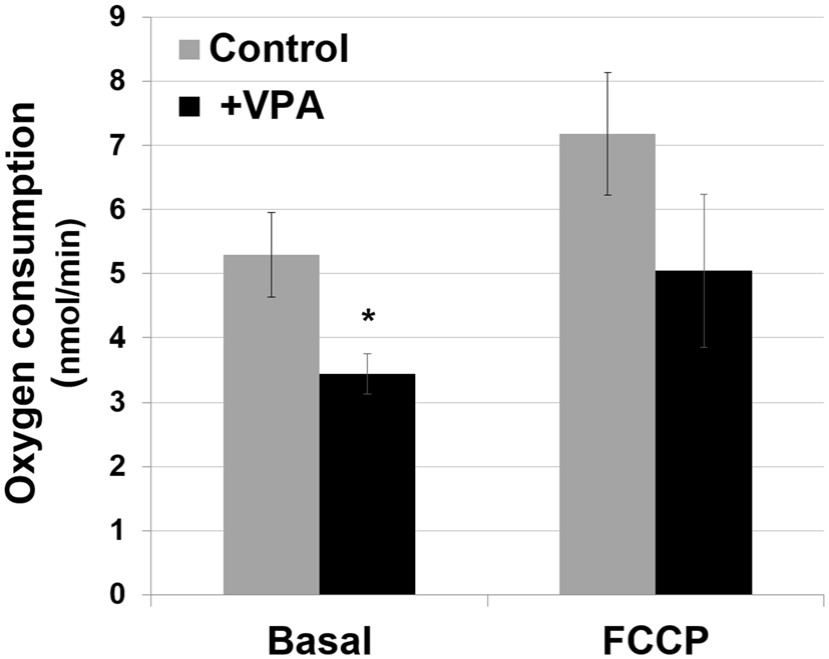
VPA decreases oxygen consumption. Respiration was measured using 5 mL of mid-log phase yeast cells with a Clark-type electrode. VPA (1 mM) or dH_2_O was added to the cells for 2 min and respiration was measured in the absence (Basal) or presence of uncoupler FCCP (5 uM) to achieve maximum respiratory capacity. Respiration rates are defined as consumed O_2_ (nmol/min) and normalized to 1 optical density unit of cells measured at A_550_. Data shown are mean ± SD (n = 3) (*p < 0.05).

One possible explanation for the observed increase in glycolysis is that it is a compensatory mechanism in response to a decrease in mitochondrial bioenergetics. To address this possibility, ΔΨ_m_ was measured using the probe tetramethylrhodamine (TMRM), a cationic fluorescent dye that accumulates in the mitochondria as a function of ΔΨ_m_. Dye fluorescence was quantified by flow cytometry. Following incubation with VPA, a significant decrease in the ΔΨ_m_ was observed, as indicated by decreased TMRM fluorescence (Fig. 4). A decrease in ΔΨ_m_ in VPA-treated cells suggested that the OCR was reduced. In agreement with this, a rapid decrease was observed in the OCR of cells treated with VPA (Fig. 5).

**Figure 6:**
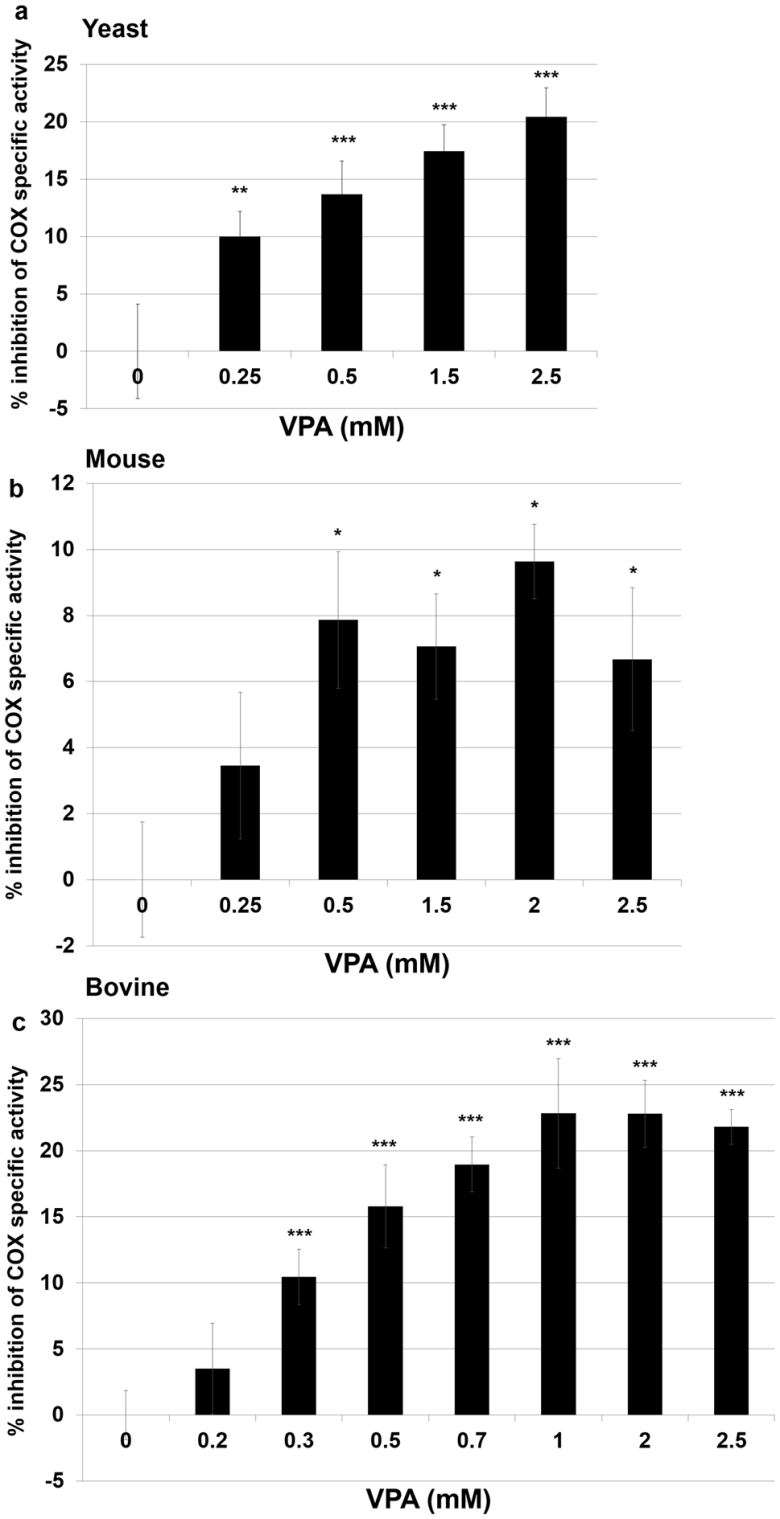
VPA inhibits COX activity. COX activity was assayed in yeast mitochondrial extracts (**a**), mouse liver homogenates (**b**), and isolated bovine liver (**c**) as described in Methods with ascorbate as a reductant using a micro Clark-type electrode in a closed chamber. COX specific activity was defined as consumed O_2_ (nmol/min/mg total protein) and reported as % inhibition relative to control. Data shown are mean ± SD (n = 4) (*p < 0.05; **p < 0.01; ***p < 0.001).

Decreased ΔΨ_m_ and OCR in response to VPA suggested that the drug may directly target COX, which catalyzes the reduction of oxygen to water and pumps protons to the mitochondrial intermembrane space, generating the ΔΨ_m_^53,54^. It has been shown that cytochrome aa_3_ depletion caused by chronic VPA administration decreases COX activity^55^. However, the decrease in OCR in this study was observed immediately after addition of the drug. To test the possibility that acute VPA inhibits COX, COX specific activity was assayed in the presence of yeast cytochrome *c*. VPA in concentrations ranging from 0.25 to 2.5 mM significantly inhibited COX activity by 10–20% (Fig. 6a).

**Figure 7:**
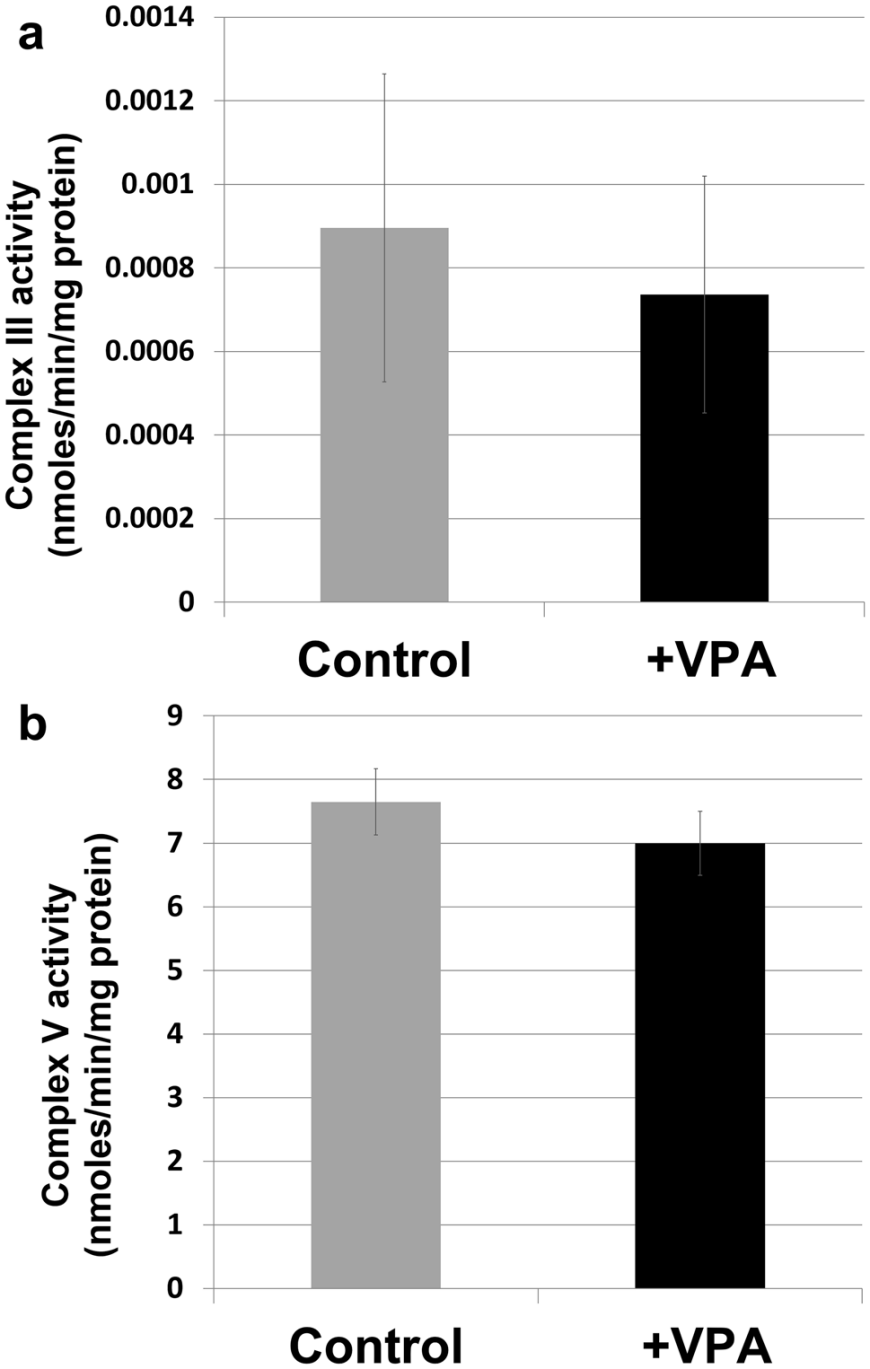
VPA does not affect complex III or complex V activities. Specific activities (nmoles/min/mg protein) of complex III (**a**) and complex V (**b**) were assayed in yeast mitochondrial extracts as described in Methods. Complex activity was assayed at two protein concentrations to ensure linearity of the reaction. Data shown are mean ± SD (n = 3 with at least two technical replicates).

A small inhibition of COX by VPA may be a contributing factor underlying perturbation of mitochondrial energy metabolism. To determine if mammalian COX, similar to the yeast enzyme, is inhibited by VPA, mouse liver homogenates were incubated with VPA at 37 °C for 25 min. Similar to the effect on the yeast enzyme, VPA significantly inhibited COX activity in mouse liver homogenates (Fig. 6b). To ascertain whether VPA inhibits COX directly, VPA was incubated with isolated bovine COX and activity was monitored. Therapeutically relevant concentrations of VPA inhibited activity of the isolated enzyme by ~ 15% (Fig. 6c). The specific activities of complex III and V were not decreased by 1 mM VPA (Fig. 7). These findings suggest that VPA binds to COX and directly inhibits its activity.

## Discussion

In this study, we show that clinically relevant concentrations of VPA increase carbon accumulation through glycolysis and decrease mitochondrial bioenergetics. Furthermore, we demonstrate for the first time that VPA directly inhibits mammalian COX activity, which contributes to inhibition of mitochondrial function by the drug.

This is the first description of a VPA-mediated increase in glycolysis (Figs. 2a, 3a). Because of its central position in metabolism, glycolysis is very active in rapidly proliferating cells such as yeast and cancer cells^56^. In addition to generating ATP, many glycolytic intermediates are used in other anabolic pathways that support active growth. For example, the pentose phosphate pathway generates ribose (for nucleic acid synthesis) and NADPH (for lipid and nucleic acid synthesis and reduction of glutathione). As Fig. 2b indicates, VPA did not affect levels of metabolites of the non-oxidative phase of the pentose phosphate pathway. Yeast cells ferment the end-product of glycolysis (pyruvate) to ethanol. Because the increase in levels of glycolytic intermediates was accompanied by an increase in ethanol production (Fig. 1), it is likely that the majority of the carbon utilized through glycolysis was converted to ethanol and not incorporated into anabolic pathways associated with cell growth and division. This is further supported by findings showing that VPA inhibited proliferation and cell cycle progression in yeast cells ^51,57^.

Activation of glycolysis by VPA is likely the response to inhibition of mitochondrial energy metabolism. VPA-treated cells exhibited a decrease in ΔΨ_m_ as indicated by a 50% reduction of TMRM fluorescence (Fig. 4). This was accompanied by a 30% decrease in OCR (Fig. 5), which occurred immediately after addition of the drug. Our findings agree with those of Komulainen et al.^48^, who reported that VPA decreased ΔΨ_m_ and OCR in HepG2 cells. Here, we show that VPA directly inhibits yeast and mammalian COX activity (Fig. 6). Maximal inhibition of mammalian COX was observed in response to 0.5 to 1 mM VPA (Fig. 6c), a concentration range present in patients’ blood during treatment^58^. VPA (1 mM) did not decrease the activities of complexes III and V (Fig. 7). COX is the terminal and proposed rate-limiting enzyme of the electron transport chain^59–64^. COX exerts tight control over cellular respiration and membrane potential, as demonstrated in intact HepG2 cells^59–61^. Pacelli and co-workers reported that even a small degree of inhibition of COX leads to a significant decrease in membrane potential^61^, which affects not only energy production but also mitochondrial protein import and calcium homeostasis. It is not surprising, therefore, that further inhibition of electron transport enzymes may have detrimental consequences, as suggested by the report of liver failure resulting from VPA treatment of a patient with a COX deficiency^32^. These findings suggest that inhibition of COX by VPA contributes to toxicity, and that VPA may be contraindicated for patients with defects in pathways related to energy metabolism. The most likely mechanism of inhibition of energy metabolism by VPA is direct inhibition of COX, although we cannot rule out additional inhibitory effects of the drug on other pathways, such as β-oxidation^35^ and the TCA cycle^41^.

In conclusion, VPA inhibits mitochondrial bioenergetics and increases glycolytic activity in yeast. Intriguingly, studies using magnetic resonance spectroscopy of brains of BD patients indicate that BD pathophysiology involves mitochondrial dysfunction and a resultant increase in glycolysis for energy production^65^. However, it is not always possible to separate the effects of the medications from the underlying pathophysiology. Therefore, VPA treatment may contribute to an increase in glycolysis in the brain. This possibility is supported by an increase in lactate production in rat brains after acute VPA^66^. Additionally, the inhibitory effect of VPA on mitochondrial metabolism could explain VPA toxicity, especially hepatotoxicity^29,30^. Alternatively, inhibition of mitochondrial bioenergetics may be a therapeutic response to VPA, as several markers of mitochondrial activity are higher in bipolar mania than in the euthymic and depressive phases of the disorder^13^. This could explain why VPA is mainly prescribed for its acute antimanic effects^67–69^. Future studies should further investigate whether the increase in glycolysis caused by VPA is therapeutically relevant, or is a contributing factor to the pathophysiology of BD.

## Methods

### Yeast strain, growth medium, and conditions

The *Saccharomyces cerevisiae* strain used in this study was BY4741 *MATa* purchased from Invitrogen. Cells were maintained on YPD medium (2% bactopeptone, 1% yeast extract, 2% glucose) and grown in synthetic minimal medium without inositol (I-), which contained all the essential components of Difco yeast nitrogen base (except inositol), 2% glucose, 0.2% ammonium sulfate, vitamin mix, histidine (20 mg/L), methionine (20 mg/L), leucine (60 mg/L) and uracil (40 mg/L). Where indicated, inositol was added at a concentration of 75 μM (I +). VPA (sodium valproate, Sigma) was added to a final concentration of either 0.6 mM or 1 mM, while controls were administered dH_2_O. Absorbance was measured at 550 nm to monitor growth in liquid cultures. All incubations were at 30 °C.

### VPA treatment

Cells were pre-cultured overnight in synthetic minimal medium with inositol (I +), harvested, washed twice with similar medium lacking inositol (I-) and resuspended in the same medium. Cells were inoculated to a final A_550_ of 0.05 and cultured until the cells reached the mid log phase (A_550_ = 0.5–0.7). VPA or dH_2_O was then added and cultures were incubated for 30 min or 5 h.

### Ethanol

Cells were treated with VPA for the specified times and pelleted. Ethanol concentrations were measured in supernatants using a fluorometric assay kit from BioVision (K620). Ethanol concentration is in mM ethanol normalized to cell density as measured at A_550_.

### Mass spectrometry of glycolytic and pentose phosphate pathway metabolite levels

Cell cultures (10 mL) were grown to the mid-log phase and treated with VPA (1 mM) or dH_2_O for 5 h. Cells were then quenched, and metabolites were extracted as described^70^. Quantification of metabolites was determined by mass spectrometry at the Karmanos Cancer Institute Pharmacology Core. The values represent the concentration of each metabolite (µM) normalized to cell density as measured at A_550_.

### [U-^13^C]-glucose metabolic labelling and mass spectrometry

Cells were grown to the mid-log phase, treated with VPA (0.6 mM) or dH_2_O for 4.5 h, harvested, and incubated in fresh medium containing uniformly labeled [U-^13^C]-glucose (Omicron Biochemicals, Inc) for 20 min. Cells were then washed with 150 mM ammonium acetate and snap frozen in liquid nitrogen. To assay carbon flux, cells were lysed, protein concentration was determined, and quantification of [U-^13^C]-labelled glycolytic and pentose phosphate pathway metabolites was determined by mass spectrometry at the University of Michigan Metabolomics Core.

### Mitochondrial membrane potential

Cells were grown to the mid-log phase and then incubated with 0.6 mM VPA and 500 nM TMRM for 1 h. Cells were washed once with dH_2_O and fluorescence was measured using flow cytometry (BD LSR II) at the Microscopy, Imaging & Cytometry Resources (MICR) Core at Wayne State University. Dye fluorescence is proportional to mitochondrial membrane potential. Median fluorescence intensity (MFI) of single cells was analyzed with the FlowJo software. Data presented are in arbitrary fluorescence units (AFUs).

### Oxygen consumption rate

Cellular respiration was measured using 5 mL of mid-log phase yeast cells (A_550_ = 0.6) with a Clark-type electrode (YSI 5300) at 30 °C. VPA (1 mM) or dH_2_O was added directly to the chamber and measurement was initiated after 2 min. Mitochondrial uncoupler FCCP (5 µM) was added to determine the maximal respiratory capacity. KCN (0.2 mM) was added at the end of the experiment to correct for non-COX oxygen consumption. Respiration rates are defined as consumed O_2_ (nmol) per min and normalized to 1 optical density unit of cells measured at A_550_.

### Yeast COX activity

Cells were grown in 1.5 L cultures of YPGal (2% bactopeptone, 1% yeast extract, 2% galactose) until the late-log phase. Cells were pelleted and mitochondria were isolated following zymolase treatment and differential centrifugation as described^71^. Isolated mitochondria were stored at -80 °C. COX activity was assayed in the presence of 20 μM *S. cerevisiae* cytochrome *c* (Sigma) and 20 mM ascorbic acid as a reductant. Briefly, mitochondria were incubated with the specified concentrations of VPA and COX activity was assayed using a micro-Clark electrode (Oxygraph system, Hansatech) at 25 °C. Oxygen consumption was recorded on a computer and analyzed with Oxygraph software. Protein concentration was determined with the DC protein assay kit (Bio-Rad, Hercules, CA, USA). COX specific activity is defined as consumed O_2_ (nmol)/min/mg total protein and reported as percentage of control.

### Mammalian COX activity

Mouse liver tissue or isolated bovine COX was incubated with VPA and COX activity was assayed as described^72,73^. Procedures for acquiring animal tissues were approved by the Wayne State University Institutional Animal Care and Use Committee (IACUC), and all experiments were performed in accordance with relevant guidelines and regulations. Briefly, mouse livers from 8-week old animals were harvested from euthanized animals, minced in incubation buffer (250 mM sucrose, 20 mM Tris buffer pH 7.4, 1 mM PMSF), and incubated at 37 °C for 25 min with different concentrations of VPA. Tissue was collected by centrifugation at 4 °C, the supernatant discarded, and samples stored frozen at − 80 °C. COX activity was measured with a micro Clark-type oxygen electrode in a closed chamber (Oxygraph system, Hansatech) at 25 °C. Frozen pellets were solubilized in 10 mM K-HEPES, 40 mM KCL, 1% Tween-20, 2 mM EGTA, 1 mM Na-vanadate, 1 mM PMSF, 1 μM oligomycin by sonication for 5 s, 2 times. The supernatant was collected, and COX activity measured in the presence of 20 μM cow heart cytochrome *c* (Sigma) and 20 mM ascorbic acid as a reductant. Oxygen consumption was recorded on a computer and analyzed with the Oxygraph software. Protein concentration was determined with the DC protein assay kit (Bio-Rad, Hercules, CA, USA). COX specific activity is defined as consumed O_2_ (nmol)/min/mg total protein and reported as percentage of control. Measurements with COX isolated from bovine liver were performed in a similar manner; see^73^ for details.

### Complex III and V specific activities

Yeast mitochondria prepared as described above were used to assay complex III and complex V specific activities as described^74^. Briefly, isolated mitochondria were resuspended in potassium phosphate buffer (10 mM), pH 7.4. Samples were exposed to three freeze–thaw cycles. Protein concentration (determined by the Bio-Rad Bradford assay) was normalized to 0.5 µg/µL. For complex III activity, aliquots corresponding to 4–8 µg protein were added to a cuvette containing 200 µL potassium phosphate buffer (250 mM), pH 7.4, 40 µL sodium azide (50 mM), and 50 µL freshly prepared cytochrome *c* (1 mM). The volume was adjusted to 990 µL with dH_2_O and the baseline was recorded at 550 nm for 2 min. The reaction was initiated by adding 10 µL chemically reduced decylubiquinol (10 mM). Absorbance of the sample was measured for 2 min. Complex III specific activity was determined by subtracting the absorbance of a parallel cuvette containing 10 µL antimycin A (1 mg/mL). VPA (1 mM) effect was assayed in parallel cuvettes incubated for 10 min at 30 °C.

To assay complex V activity, aliquots corresponding to 4–8 µg protein were added to a cuvette containing 500 µL magnesium sulfate (10 mM) in 100 mM Hepes–KOH, pH 8.0, 10 µL NADH (30 mM), 50 µL phosphoenolpyruvic acid (50 mM), 5 µL pyruvate kinase (10 mg/mL), 10 µL lactate dehydrogenase (5 mg/mL), and 10 µL antimycin A (0.2 mg/mL). The volume was adjusted to 900 µL with dH_2_O and the baseline was recorded at 340 nm for 2 min. The reaction was initiated by adding 100 µL ATP (25 mM). Absorbance was measured for 2 min. 10 µL oligomycin (0.2 mg/mL) was then added and the measurement resumed for another 2 min. The measurement in the presence of oligomycin was subtracted to determine complex V specific activity. VPA (1 mM) effect was assayed in parallel cuvettes incubated for 10 min at 30 °C.

Specific activities were determined for protein concentrations that ensured the linearity of the reaction and calculated using the Beer-Lambert law equation^74^. Activities are expressed as nmoles/min/mg protein.

## Statistical analysis

All significance values were calculated by the two sample Student’s *t* test.

## Data Availability

All data generated or analyzed during this study are included in this published article.

## References

[1] Bostwick JM, Pankratz VS (2000). Affective disorders and suicide risk: a reexamination. Am. J. Psychiatry.

[2] Mansur RB, Brietzke E (2012). The, "selfish brain" hypothesis for metabolic abnormalities in bipolar disorder and schizophrenia. Trends Psychiatry Psychother..

[3] Silarova B (2015). Metabolic syndrome in patients with bipolar disorder: comparison with major depressive disorder and non-psychiatric controls. J Psychosom. Res..

[4] Johnson CP (2015). Brain abnormalities in bipolar disorder detected by quantitative T1rho mapping. Mol. Psychiatry.

[5] Li CT (2015). Peripheral and central glucose utilizations modulated by mitochondrial DNA 10398A in bipolar disorder. Psychoneuroendocrinology.

[6] Hamakawa H (2004). Reduced intracellular pH in the basal ganglia and whole brain measured by 31P-MRS in bipolar disorder. Psychiatry Clin. Neurosci..

[7] Kato T (1998). Decreased brain intracellular pH measured by 31P-MRS in bipolar disorder: a confirmation in drug-free patients and correlation with white matter hyperintensity. Eur. Arch. Psychiatry Clin. Neurosci..

[8] Dager SR (2004). Brain metabolic alterations in medication-free patients with bipolar disorder. Arch. Gen. Psychiatry.

[9] Kato T (1995). Lateralized abnormality of high energy phosphate metabolism in the frontal lobes of patients with bipolar disorder detected by phase-encoded 31P-MRS. Psychol. Med..

[10] Bertolino A (2003). Neuronal pathology in the hippocampal area of patients with bipolar disorder: a study with proton magnetic resonance spectroscopic imaging. Biol. Psychiat..

[11] Chang K (2003). Decreased N-acetylaspartate in children with familial bipolar disorder. Biol. Psychiat..

[12] Deicken RF, Pegues MP, Anzalone S, Feiwell R, Soher B (2003). Lower concentration of hippocampal N-acetylaspartate in familial bipolar I disorder. Am. J. Psychiatry.

[13] Morris G (2017). A model of the mitochondrial basis of bipolar disorder. Neurosci. Biobehav. Rev..

[14] Caliyurt O, Altiay G (2009). Resting energy expenditure in manic episode. Bipolar Disord..

[15] Baxter LR (1985). Cerebral metabolic rates for glucose in mood disorders. Studies with positron emission tomography and fluorodeoxyglucose F 18. Arch. Gen. Psychiatry.

[16] Albert U (2015). Increased uric acid levels in bipolar disorder subjects during different phases of illness. J. Affect. Disord..

[17] Dubovsky SL, Daurignac E, Leonard KE (2014). Increased platelet intracellular calcium ion concentration is specific to bipolar disorder. J. Affect. Disord..

[18] Cipriani A, Reid K, Young AH, Macritchie K, Geddes J (2013). Valproic acid, valproate and divalproex in the maintenance treatment of bipolar disorder. Cochrane Database Syst Rev..

[19] Kessing LV, Hellmund G, Geddes JR, Goodwin GM, Andersen PK (2011). Valproate v lithium in the treatment of bipolar disorder in clinical practice: observational nationwide register-based cohort study. Br. J. Psychiatry.

[20] Brown EG (1986). Sodium valproate in epilepsy treatment. Practitioner.

[21] Petrukhin AS, Mukhin K (2001). Depakene (sodium valproate) in the treatment of epilepsy in children and adolescents: efficiency and safety. Zh Nevrol Psikhiatr Im S S Korsakova.

[22] Blumenfeld A, Gennings C, Cady R (2012). Pharmacological synergy: the next frontier on therapeutic advancement for migraine. Headache.

[23] Gould TD, Quiroz JA, Singh J, Zarate CA, Manji HK (2004). Emerging experimental therapeutics for bipolar disorder: insights from the molecular and cellular actions of current mood stabilizers. Mol. Psychiatry.

[24] Henry TR (2003). The history of valproate in clinical neuroscience. Psychopharmacol. Bull..

[25] Lewis JH, Zimmerman HJ, Garrett CT, Rosenberg E (1982). Valproate-induced hepatic steatogenesis in rats. Hepatology.

[26] Lee MH (2007). Gene expression profiles of murine fatty liver induced by the administration of valproic acid. Toxicol. Appl. Pharmacol..

[27] Schmid MM (2013). Non-fatal and fatal liver failure associated with valproic acid. Pharmacopsychiatry.

[28] Scheffner D (1988). Fatal liver failure in 16 children with valproate therapy. Epilepsia.

[29] Konig SA (1999). Fatal liver failure associated with valproate therapy in a patient with Friedreich's disease: review of valproate hepatotoxicity in adults. Epilepsia.

[30] Zhang LF (2014). Combined effects of a high-fat diet and chronic valproic acid treatment on hepatic steatosis and hepatotoxicity in rats. Acta Pharmacol. Sin..

[31] Begriche K, Igoudjil A, Pessayre D, Fromenty B (2006). Mitochondrial dysfunction in NASH: causes, consequences and possible means to prevent it. Mitochondrion.

[32] Chabrol B (1994). Valproate-induced hepatic failure in a case of cytochrome c oxidase deficiency. Eur. J. Pediatr..

[33] Krahenbuhl S, Brandner S, Kleinle S, Liechti S, Straumann D (2000). Mitochondrial diseases represent a risk factor for valproate-induced fulminant liver failure. Liver.

[34] Lam CW, Lau CH, Williams JC, Chan YW, Wong LJ (1997). Mitochondrial myopathy, encephalopathy, lactic acidosis and stroke-like episodes (MELAS) triggered by valproate therapy. Eur. J. Pediatr..

[35] Silva MF (2008). Valproic acid metabolism and its effects on mitochondrial fatty acid oxidation: a review. J. Inherit. Metab. Dis..

[36] Becker CM, Harris RA (1983). Influence of valproic acid on hepatic carbohydrate and lipid metabolism. Arch. Biochem. Biophys..

[37] Ito M, Ikeda Y, Arnez JG, Finocchiaro G, Tanaka K (1990). The enzymatic basis for the metabolism and inhibitory effects of valproic acid: dehydrogenation of valproyl-CoA by 2-methyl-branched-chain acyl-CoA dehydrogenase. Biochem. Biophys. Acta..

[38] Baldwin GS, Abbott FS, Nau H (1996). Binding of a valproate metabolite to the trifunctional protein of fatty acid oxidation. FEBS Lett..

[39] Johannessen CU, Petersen D, Fonnum F, Hassel B (2001). The acute effect of valproate on cerebral energy metabolism in mice. Epilepsy Res..

[40] Luder AS, Parks JK, Frerman F, Parker WD (1990). Inactivation of beef brain alpha-ketoglutarate dehydrogenase complex by valproic acid and valproic acid metabolites. Possible mechanism of anticonvulsant and toxic actions. J. Clin. Investig..

[41] El Hage M, Baverel G, Martin G (2012). Effects of valproate on glutamate metabolism in rat brain slices: a (13)C NMR study. Epilepsy Res..

[42] Ohtani Y, Endo F, Matsuda I (1982). Carnitine deficiency and hyperammonemia associated with valproic acid therapy. J. Pediatr..

[43] Raskind JY, El-Chaar GM (2000). The role of carnitine supplementation during valproic acid therapy. Ann. Pharmacother..

[44] Silva MF (1997). Valproate inhibits the mitochondrial pyruvate-driven oxidative phosphorylation in vitro. J. Inherit. Metab. Dis..

[45] Aires CC (2008). Pyruvate uptake is inhibited by valproic acid and metabolites in mitochondrial membranes. FEBS Lett..

[46] Haas R, Stumpf DA, Parks JK, Eguren L (1981). Inhibitory effects of sodium valproate on oxidative phosphorylation. Neurology.

[47] Rumbach L (1983). Inhibition of oxidative phosphorylation in hepatic and cerebral mitochondria of sodium valproate-treated rats. J. Neurol. Sci..

[48] Komulainen T (2015). Sodium valproate induces mitochondrial respiration dysfunction in HepG2 in vitro cell model. Toxicology.

[49] Deranieh RM (2015). Perturbation of the Vacuolar ATPase: a novel consequence of inositol depletion. J. Biol. Chem..

[50] Jadhav S (2016). Valproate induces the unfolded protein response by increasing ceramide levels. J. Biol. Chem..

[51] Golla U, Joseph D, Tomar RS (2016). Combined transcriptomics and chemical-genetics reveal molecular mode of action of valproic acid, an anticancer molecule using budding yeast model. Sci. Rep..

[52] Mitsui K, Nakagawa D, Nakamura M, Okamoto T, Tsurugi K (2005). Valproic acid induces apoptosis dependent of Yca1p at concentrations that mildly affect the proliferation of yeast. FEBS Lett..

[53] Brunori M, Giuffre A, Sarti P (2005). Cytochrome c oxidase, ligands and electrons. J. Inorg. Biochem..

[54] Brzezinski P, Gennis RB (2008). Cytochrome c oxidase: exciting progress and remaining mysteries. J. Bioenergy Biomembr..

[55] Ponchaut S, van Hoof F, Veitch K (1992). Cytochrome aa3 depletion is the cause of the deficient mitochondrial respiration induced by chronic valproate administration. Biochem. Pharmacol..

[56] Diaz-Ruiz R, Rigoulet M, Devin A (1807). The Warburg and Crabtree effects: On the origin of cancer cell energy metabolism and of yeast glucose repression. Biochem. Biophys. Acta..

[57] Desfosses-Baron K (2016). Valproate inhibits MAP kinase signalling and cell cycle progression in *S cerevisiae*. Sci. Rep..

[58] Lindberger M, Tomson T, Stahle L (2003). Unbound valproate fraction in plasma and subcutaneous microdialysate in steady state and after a single dose in humans. Ther. Drug Monit..

[59] Acin-Perez R (2003). An intragenic suppressor in the cytochrome c oxidase I gene of mouse mitochondrial DNA. Hum. Mol. Genet.

[60] Dalmonte ME (2009). Control of respiration by cytochrome c oxidase in intact cells: role of the membrane potential. J. Biol. Chem..

[61] Pacelli C (2011). Tight control of mitochondrial membrane potential by cytochrome c oxidase. Mitochondrion.

[62] Piccoli C, Scrima R, Boffoli D, Capitanio N (2006). Control by cytochrome c oxidase of the cellular oxidative phosphorylation system depends on the mitochondrial energy state. Biochem. J..

[63] Villani G, Attardi G (1997). In vivo control of respiration by cytochrome c oxidase in wild-type and mitochondrial DNA mutation-carrying human cells. Proc. Natl. Acad. Sci. USA.

[64] Villani G, Greco M, Papa S, Attardi G (1998). Low reserve of cytochrome c oxidase capacity in vivo in the respiratory chain of a variety of human cell types. J. Biol. Chem..

[65] Stork C, Renshaw PF (2005). Mitochondrial dysfunction in bipolar disorder: evidence from magnetic resonance spectroscopy research. Mol. Psychiatry.

[66] Gueldry S, Rochette L, Bralet J (1987). Comparison of the effects of valproate, ethosuximide, phenytoin, and pentobarbital on cerebral energy metabolism in the rat. Epilepsia.

[67] American Psychiatric A (2002). Practice guideline for the treatment of patients with bipolar disorder (revision). Am. J. Psychiatry.

[68] Goodwin, G. M., & Consensus Group of the British Association for P (2009). Evidence-based guidelines for treating bipolar disorder: revised second edition—recommendations from the British Association for Psychopharmacology. J. Psychopharmacol..

[69] Yatham LN (2013). Canadian Network for Mood and Anxiety Treatments (CANMAT) and International Society for Bipolar Disorders (ISBD) collaborative update of CANMAT guidelines for the management of patients with bipolar disorder: update 2013. Bipolar Disord..

[70] Gonzalez B, Francois J, Renaud M (1997). A rapid and reliable method for metabolite extraction in yeast using boiling buffered ethanol. Yeast.

[71] Altmann K, Durr M, Westermann B (2007). Saccharomyces cerevisiae as a model organism to study mitochondrial biology: general considerations and basic procedures. Methods Mol. Biol..

[72] Lee I (2009). Isolation of regulatory-competent, phosphorylated cytochrome C oxidase. Methods Enzymol..

[73] Lee I (2005). cAMP-dependent tyrosine phosphorylation of subunit I inhibits cytochrome c oxidase activity. J. Biol. Chem..

[74] Magri S, Fracasso V, Rimoldi M, Taroni F (2010). Preparation of yeast mitochondria and in vitro assay of respiratory chain complex activities. Nat. Protoc..

